# Accuracy of Attestation Among Micrographic Dermatologic Surgery Diplomates

**DOI:** 10.1001/jamanetworkopen.2022.29795

**Published:** 2022-09-02

**Authors:** Clifford S. Perlis, Roy H. Perlis

**Affiliations:** 1 Keystone Dermatology Partners, Willow Grove, Pennsylvania; 2 Center for Quantitative Health and Department of Psychiatry, Massachusetts General Hospital and Harvard Medical School, Boston, Massachusetts; 3 Associate Editor, *JAMA Network Open*

## Abstract

This quality improvement study examines the standards of certification by the American Board of Dermatology for performance of micrographic dermatologic surgery.

## Introduction

The American Board of Dermatology (ABD) created a subspecialty certification for micrographic dermatologic surgery (MDS) and announced diplomates on December 14, 2021. Certification required successful completion of an examination given in October 2021, a current medical license, primary ABD certification in dermatology, and current Maintenance of Certification. Candidates also had to demonstrate experience in MDS through completion of an Accreditation Council for Graduate Medical Education (ACGME) fellowship or “attesting to active practice of micrographic surgery.”^1^ The ABD did not define or require documentation of active practice. We assessed the self-regulatory process.

## Methods

Using public data in this quality improvement study, we assessed how many MDS diplomates actively practice Mohs surgery (ie, perform an average of 1 case per week or 50 cases per year). To identify such physicians, we compared the list of diplomates on the ABD website^2^ with the 2019 Centers for Medicare & Medicaid Services (CMS) billing data.^3^ Some diplomates may perform Mohs surgery but may not have billed CMS in 2019. To ensure our list captured diplomates who billed CMS in 2019, we included only individuals with charges for codes *Common Procedural Terminology *(*CPT*) 17000 or 11102 (destruction premalignant lesion and shave biopsy), assuming billing for 1 of these codes would have occurred at least once per year. Any diplomate with no such charges was excluded. Per the Common Rule (45 CFR §46), institutional review board approval was not sought because this study was based on deidentified data that are publicly available. This study followed the SQUIRE reporting guideline.

## Results

Of 1716 MDS diplomates, we eliminated physicians who could not be matched to a National Provider Identifier (NPI) number (335), had multiple NPI numbers (36), or lacked charges for *CPT* 11102 or 17000 (63). The high number not matched to an individual NPI number could be the result of billing under a type 2 (group) NPI, working for an organization that did not require an NPI, or an unidentified situation. The remaining 1282 individuals (74.7%) were stratified based on the number of times they billed for Mohs surgery (*CPT* 17311 or 17313); 1109 individuals had charged for greater than 50 cases. Completing an ACGME-accredited MDS fellowship also qualifies individuals to take the examination. The ACGME does not maintain a list of fellows. We eliminated 48 individuals who were listed in the American College of Mohs Surgery 2021-2022 membership directory and 14 individuals who listed completion of an ACGME-accredited fellowship on their own websites. This yielded 111 physicians who achieved MDS board certification without completing a fellowship or averaging more than 1 Mohs case per week. Among them, 72 did not charge for any Mohs procedures (Figure).

**Figure.**
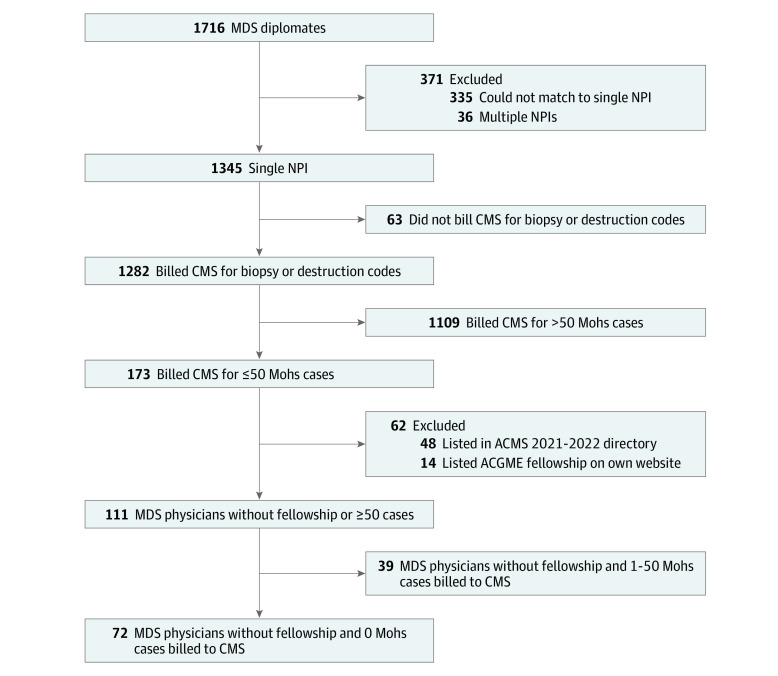
Micrographic Dermatologic Surgery (MDS) Diplomates ACGME indicates Accreditation Council for Graduate Medical Education; ACMS, American College of Mohs Surgery; CMS, Centers for Medicare & Medicaid Services; and NPI, National Provider Identifier.

## Discussion

Study limitations include some diplomates may actively practice Mohs surgery but not bill CMS, individuals may disagree with our definition for active practice of Mohs surgery, and some diplomates may have completed fellowships that we were unable to identify.

Our assumptions may underestimate the number of individuals who achieved MDS certification without actively practicing Mohs surgery. More than 21% of diplomates (371) were eliminated because we were unable to match each physician to a single NPI number. Furthermore, 63 individuals were eliminated because they did not bill for either *CPT* 17000 or 11102.

Before introduction of MDS certification, the most recently introduced subspecialty certification in dermatology was for pediatric dermatology. Grandfathering allowed physicians without fellowship training to take the examination if they had 5 or more years of clinical dermatology experience with at least half of their caseloads being pediatric. Furthermore, these individuals had to apply using supporting publications, lectures, and letters of reference for case-by-case consideration of eligibility.^4^

Professional organizations granting board certification protect the public through identifying physicians who have achieved certain levels of expertise. Greater than 6% of new diplomates did not charge CMS for more than 50 Mohs cases in 2019, including 72 with no record of charging CMS for any Mohs cases. This discrepancy between the test qualifications and practice patterns evident from CMS data suggests concern for the reliability of self-regulation by attesting physicians and the ABD.
